# Modulation of phosphofructokinase (PFK) from *Setaria cervi*, a bovine filarial parasite, by different effectors and its interaction with some antifilarials

**DOI:** 10.1186/1756-3305-4-227

**Published:** 2011-12-07

**Authors:** Bechan Sharma

**Affiliations:** 1 Department of Biochemistry, Faculty of Science, University of Allahabad, Allahabad-211002, UP, India

**Keywords:** Phosphofructokinase, *Setaria cervi*, Nucleotides, Specificity, Activation, Inhibition, Antifilarials

## Abstract

**Background:**

Phosphofructokinase (ATP: D-fructose-6-phosphate-1-phosphotransferase, EC 2.7.1.11, PFK) is of primary importance in the regulation of glycolytic flux. This enzyme has been extensively studied from mammalian sources but relatively less attention has been paid towards its characterization from filarial parasites. Furthermore, the information about the response of filarial PFK towards the anthelmintics/antifilarial compounds is lacking. In view of these facts, PFK from *Setaria cervi*, a bovine filarial parasite having similarity with that of human filarial worms, was isolated, purified and characterized.

**Results:**

The *S. cervi *PFK was cytosolic in nature. The adult parasites (both female and male) contained more enzyme activity than the microfilarial (Mf) stage of *S. cervi*, which exhibited only 20% of total activity. The *S. cervi *PFK could be modulated by different nucleotides and the response of enzyme to these nucleotides was dependent on the concentrations of substrates (F-6-P and ATP). The enzyme possessed wide specificity towards utilization of the nucleotides as phosphate group donors. *S. cervi *PFK showed the presence of thiol group(s) at the active site of the enzyme, which could be protected from inhibitory action of para-chloromercuribenzoate (p-CMB) up to about 76% by pretreatment with cysteine or β-ME. The sensitivity of PFK from *S. cervi *towards antifilarials/anthelmintics was comparatively higher than that of mammalian PFK. With suramin, the Ki value for rat liver PFK was 40 times higher than PFK from *S. cervi*.

**Conclusions:**

The results indicate that the activity of filarial PFK may be modified by different effectors (such as nucleotides, thiol group reactants and anthelmintics) in filarial worms depending on the presence of varying concentrations of substrates (F-6-P and ATP) in the cellular milieu. It may possess thiol group at its active site responsible for catalysis. Relatively, 40 times higher sensitivity of filarial PFK towards suramin as compared to the analogous enzyme from the mammalian system indicates that this enzyme could be exploited as a potential chemotherapeutic target against filariasis.

## Background

Although considerable research has been done in the field of morphology, life cycle and taxonomy of filarial parasites, comparatively little attention has been paid to the physiology and metabolism of the filarial worms and their effects on the host. The basic stumbling block in the design of suitable antifilarial drugs is beset with our poor knowledge about the metabolic activities of adult and various developmental stages of filarial worms as well as the disorders generated in the host harbouring the infection. The non-availability of experimental materials from human filarial parasites and insignificant progress made in culturing them under *in vitro *condition, have further precluded their study [1].

*Setaria cervi*, a bovine filarial parasite, dwelling in the lymphatics and intraperitoneal folds of naturally infected Indian water buffaloes (*Bubalus bubalis *Linn.), serves as a unique experimental model for such studies as it resembles human filarial worms in nocturnal periodicity, metabolic pathways, antigenic make up and sensitivity towards antifilarials, and anthelmintic compounds. Furthermore, this worm may be obtained in sufficient quantity from any local abattoir for carrying out enzyme purification and desired experiments towards detailed characterization [2-4].

Phosphofructokinase (ATP: D-fructose-6-phospho-1-phosphotransferase, EC 2.7.1.11, PFK) is a key enzyme which is responsible for catalyzing the transfer of the terminal phosphate of ATP to the C-1 hydroxyl group of Fructose-6-phosphate (F-6-P) to produce

fructose-1,6-diphosphate (FDP). Since, many of the parasites in general and filarial parasites in particular utilize glycolysis as a major source of energy for their survival, the study of this enzyme becomes highly pertinent [2,4-8]. Filarial worms do not catalyze the complete oxidation of the substrate to CO_2 _and reduced organic acids as end product of the metabolism [2,6,7,9]. The filarial nematodes are known to utilize a limited quantity of oxygen, when available and possess rudimentary and unusual electron transport chains that catalyze limited terminal oxidation with generation of little energy [2,6,10,11].

Earlier reports have indicated comparatively low activity of PFK in *S. cervi *suggesting thereby that this enzyme may be playing a regulatory role in controlling the operation of the glycolytic pathway [2]. Because of the multiplicity of modifiers, PFK has served as a model in studies of allosteric regulation of enzymes. The enzyme activity appears to be modulated to meet the metabolic needs of the cell, with the metabolites serving as intracellular indicators [12-16]. Although PFK from several parasite and vertebrate sources has been purified and characterized, the information about the regulation of filarial PFK by nucleotides is not well understood. Some of the kinetic characteristics of purified PFK from *S. cervi *have already been studied and the same have been compared with the analogous enzyme isolated from the mammalian systems [2,17]. The differences in the kinetic properties of PFK from filarial worms and the mammalian sources indicated that this enzyme could be used as a potential target for design and development of suitable chemotherapeutics against filariasis.

Earlier we reported that this enzyme possesses two different pH optima depending on ATP concentrations, the values being 8.0 at low (0.1 mM) concentration which decreases to pH 7.4 at high ATP (> 0.1 mM) concentration [2]. These results indicated that the activity of filarial PFK was possibly under regulation of ATP levels [14,16,17]. The present paper illustrates the influence of different effectors including some nucleotides, thiol group reactants and anthelmintics on the kinetic characteristics of PFK purified from *S. cervi*. The results indicate that the nucleotides under different assay conditions modulate the enzyme activity differently. Also, the sensitivity of filarial PFK towards antifilarials/anthelmintics radically differs from that of mammalian liver PFK.

## Results

### Sub-cellular localization of activity of *S. cervi *PFK

In order to ascertain the sub-cellular localization of the activity of PFK from *S. cervi*, the enzyme was assayed in different sub-cellular fractions of the homogenate of the parasite. The results indicated the presence of maximum enzyme activity (up to 83%) into the cytosolic fraction of the adult female parasite (Table 1). A comparison of PFK activity in adult and microfilarial (Mf) stages of *S. cervi *indicated that adult worms (female/male) showed more activity than Mf of *S. cervi *(Table 2). However, the distribution pattern of PFK activity in the intact adult female, uteri-free adult female and Mf recovered from gravid females after microdissection indicated that only 20% of the enzyme activity could be recovered in Mf. Thus it appears that in the adult female, most of the enzyme activity is localized in the musculature (Table 3).

**Table 1:** Subcellular localization of activity of PFK in adult female *S.cervi*

Fractions	Total Protein (mg)	% Recovery of protein	Total PFK activity (Units)	% Recovery of PFK activity	Specific Activity (Units/mg protein)
1000×g	115	100	2.69	100	0.023

10,000×g	90	78	2.48	92	0.028

105,000×g	70	61	2.24	83	0.033

The activity of *S. cervi *PFK was assayed as described in Materials and Methods. The above experiments were carried out using 2.2 g of adult female *S. cervi*.

**Table 2:** Level of PFK activity in adult female, male and Mf stages of S.*cervi*

Stage of *S. cervi*	Total Protein (mg/g wet weight)	PFK activity (Units/g wet weight)	Specific Activity (Units/mg protein)
Female	40.9	2.69	0.023

Male	25.4	2.48	0.028

Mf	20.0	2.24	0.033

The activity of *S. cervi *PFK was assayed as described in Materials and Methods. In each case, PFK was assayed in the corresponding 10,000×g supernatant.

**Table 3:** Distribution of PFK from in intact adult, uteri-free female *S.cervi *and Mf

Stage of *S. cervi*	PFK activity (Units/g wet weight)	% Recovery	Specific Activity (Units/mg protein)
Female	1.13	100	0.028

Uteri-free Female	0.86	76.3	0.023

Mf	0.23	20.4	0.016

The activity of *S. cervi *PFK was assayed as described in Materials and Methods. In each case, PFK was assayed in the corresponding 10,000×g supernatant.

### Nucleotide specificity of *S. cervi *PFK

Several nucleotides di- and tri-phosphates have been studied as phosphate group donors in the phosphorylation of F-6-P catalyzed by PFK of *S. cervi*. The experiments were carried out at high and fixed concentrations of Mg^2+ ^as well as F-6-P (3.3 mM each) using purified preparation of *S. cervi *PFK. Other concentrations (except ATP) and conditions were the same as those described for the standard assay. The results are displayed in Table 4. Two concentrations of the phosphate donors (0.2 and 2.0 mM) were used. The ratio of the activity observed at 2.0 mM to that observed at 0.2 mM donor concentration provided information about possible inhibition of the enzyme at higher donor concentrations. At low concentration (0.2 mM), UTP, GTP and ADP were found to be best phosphate group donors. Among these, GTP showed the strongest inhibition at a higher concentration (2.0 mM). Other nucleotides (GDP and IDP) tested were rather poor phosphate group donors. UTP, ADP and IDP were also less inhibitory at higher concentrations. IDP showed higher activity at 2.0 mM than at 0.2 mM concentration (Table 4). These results showed that *S. cervi *PFK has a wide specificity for various nucleotides as phosphate group donors and the influence of different nucleotides on the activity of filarial PFK was reflected in a concentration dependent manner.

**Table 4:** Specificity of PFK from *S.cervi *towards different phosphate group donors

Nucleotide concentrations	Relative activity*		
	
	0.2 mM	2.0 mM	V_2.0_/V_0.2_^#^
ATP	100	22	0.22

GTP	140	10	0.071

UTP	146	58	0.40

ADP	64	36	0.55

GDP	28	12	0.43

IDP	20	34	1.67

Other concentrations and conditions were same as described for standard assay in Materials and Methods. *All values are expressed as a percentage of the activity observed at 0.2 mM ATP. # Ratio of activity observed at 2.0 mM to that observed at 0.2 mM phosphate group donor concentration.

### Nucleotides modulate the kinetics of *S. cervi *PFK

**Figure 1:**
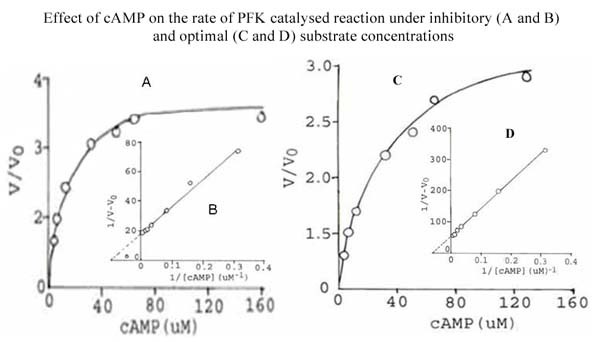
**Effect of cAMP on the rate of PFK catalyzed reaction under inhibitory (A and B) and optimal (C and D) substrate concentrations**. **Figure 1A**. Effect of cAMP on the rate of *S. cervi *PFK catalyzed reaction at fixed inhibitory concentration of ATP (1.0 mM) and low concentration of F-6-P (0.5 mM). Mg^2+ ^concentration was constant (3.3 mM). Enzyme concentration was 6.6 μg/ml. Other conditions were the same as in standard enzyme assay. V and V_0 _are rates of reaction in the presence and absence of cAMP. **Figure 1B**. Double reciprocal plot of the data of Figure 1A. **Figure 1C**. Effect of cAMP on the rate of *S. cervi *PFK catalyzed reaction at optimal concentrations of substrates (F-6-P, 3.3 mM; ATP, 0.10 mM). Mg^2+ ^concentration was constant (3.3 mM). Enzyme concentration was 6.6 μg/ml. Other conditions were the same as in standard enzyme assay. V and V_0 _are rates of reaction in the presence and absence of cAMP. **Figure 1D**. Double reciprocal plot of the data of Figure 1C.

**Figure 2:**
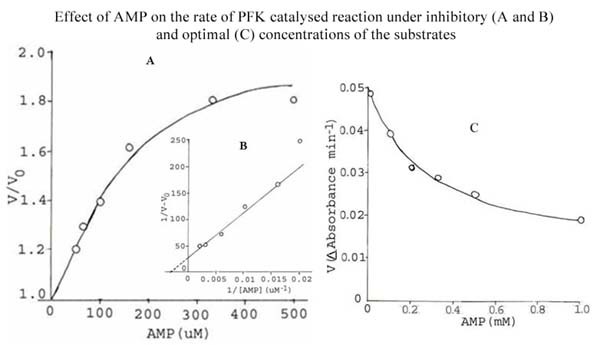
**Effect of AMP on the rate of PFK catalyzed reaction under inhibitory (A and B) and optimal (C) substrate concentrations**. **Figure 2A**. Effect of AMP on the rate of *S. cervi *PFK catalyzed reaction at fixed inhibitory concentration of ATP (1.0 mM) and low concentration of F-6-P (0.5 mM). Mg^2+ ^concentration was constant (3.3 mM). Enzyme concentration was 6.6 μg/ml. Other conditions were the same as in standard enzyme assay. V and V_0 _are rates of reaction in the presence and absence of AMP. **Figure 2B**. Double reciprocal plot of the data of Figure 2A. **Figure 2C**: Effect of AMP on the rate of *S. cervi *PFK catalyzed reaction at optimal concentrations of substrates (F-6-P, 3.3 mM; ATP, 0.10 mM). Mg^2+ ^concentration was constant (3.3 mM). Enzyme concentration was 10.0 μg/ml. Other conditions were the same as in standard enzyme assay.

**Figure 3:**
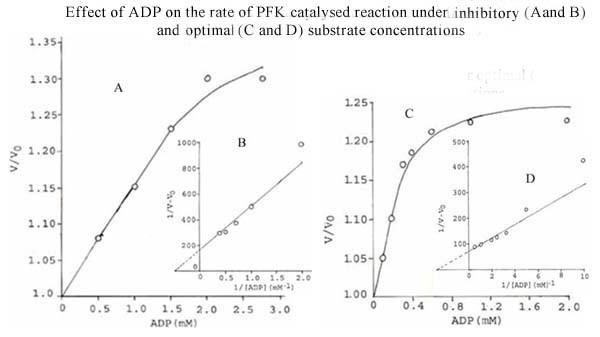
**Effect of ADP on the rate of PFK catalysed reaction under inhibitory (A and B) and optimal (C and D) substrate concentrations**. **Figure 3A**. Effect of ADP on the rate of *S. cervi *PFK catalyzed reaction at fixed inhibitory concentration of ATP (1.0 mM) and low concentration of F-6-P (0.5 mM). Mg^2+ ^concentration was constant (3.3 mM). Enzyme concentration was 6.6 μg/ml. Other conditions were the same as in standard enzyme assay. V and V_0 _are rates of reaction in the presence and absence of ADP. **Figure 3B**. Double reciprocal plot of the data of Figure 3A. **Figure 3C**. Effect of ADP on the rate of *S. cervi *PFK catalyzed reaction at optimal concentrations of substrates (F-6-P, 3.3 mM; ATP, 0.10 mM). Mg^2+ ^concentration was constant (3.3 mM). Enzyme concentration was 6.6 μg/ml. Other conditions were the same as in standard enzyme assay. V and V_0 _are rates of reaction in the presence and absence of ADP. **Figure 3D**. Double reciprocal plot of the data of Figure 3C.

Regulation of PFK activity by adenine nucleotides is well documented [18]. The effect of some nucleotides such as cAMP, AMP and ADP, on the activity of *S. cervi *PFK was studied using two different sets of conditions i.e. (1) inhibitory concentration of ATP (1.0 mM) and low concentration of F-6-P (0.5 mM) and (2) optimal concentrations of both the substrates (ATP, 0.1 mM; F-6-P, 3.3 mM). The results are shown in Figures 1, 2 and 3.

cAMP, AMP and ADP activate the enzyme at the inhibitory concentration of ATP and low concentration of F-6-P (Figures 1A, 2A and 3A). The nucleotide concentrations required for showing half (50%) maximal activation (K_A_) were found to be 0.1, 0.29 and 2.0 mM for cAMP, AMP and ADP, respectively (Inset of Figures 1B, 2B and 3B).

When the enzyme was assayed at the optimal concentration of substrates (conditions 2), only cAMP and ADP could activate *S. cervi *PFK, whereas AMP showed inhibitory effect (Figures 1C, 2C and 3C). The activation constants (K_A_) for cAMP and ADP as determined from the Figures (Figures 1D and 3D) were 0.02 and 0.36 mM, respectively. These values are close to the activation constants given above for these compounds. A summary of the results obtained under the two sets of conditions are shown in Table 5.

**Table 5:** Effect of cAMP, AMP and ADP on the activity of *S.cervi *PFK under different conditions

Effector Concentration (mM)	Experimental Conditions	% of control
cAMP (0.064)	1	348

AMP (0.38)	1	181

ADP (2.50)	1	130

cAMP (0.064)	2	282

AMP (0.48)	2	50

ADP (0.50)	2	122

Experimental condition 1: Enzyme activity was assayed against inhibitory concentrations of ATP (1.0 mM) and low concentration of F-6-P (0.5 mM). MgCl_2 _concentration was 3.3 mM in all the experiments. Experimental condition 2: Enzyme activity was assayed by the standard procedure using excess F-6-P (3.3 mM), MgCl_2 _(3.3 mM) and optimum level of ATP (0.1 mM).

### Effect of thiols and p-chloromercuribenzoate on the activity of *S. cervi *PFK

Effect of addition of thiols (cysteine and β-mercaptoethanol) and an SH-reagent, p-chloromercuribenzoate (p-CMB), has been studied on the activity of PFK from *S. cervi*. Results depicted in Table 6 showed that both cysteine and β-mercaptoethanol activated the enzyme to some extent, while p-CMB exhibited an inhibitory effect. The inhibition was partially reversed on the addition of β-ME. These data suggest that some SH-groups of the enzyme may be involved in the catalytic reaction.

**Table 6:** Effect of thiols and p-chloromercuribenzoate (p-CMB) on the activity of PFK from *S.cervi*

Compounds	Concentration (mM)	Activity remaining (%)
Control	-	100

β -ME	5	100
	
	10	170

Cysteine	5	107
	
	10	170

p-CMB	0.1	25
	
	5.0	0.2

*p-CMB+β-ME	0.1+5.0	70

#β-ME + p-CMB	5.0+0.1	100

The enzyme was pre-incubated with the effectors at 37°C for 15 min and then the activity was determined in the usual way as mentioned in Materials and Methods. *After 15 min of incubation of the enzyme with p-MB, β-ME was added, incubated for another 15 min and the reaction activity was monitored. # After 15 min incubation of the enzyme with β-ME, p-CMB was added, incubated for further 15 min and then the residual activity was monitored. Control refers to the activity in absence of any effectors.

### Effect of some antifilarial compounds on the activity of *S. cervi *PFK

**Figure 4:**
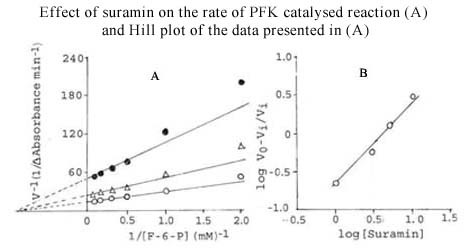
**Effect of suramin on the rate of *S. cervi *PFK-catalyzed reaction (A) and the Hill plot of the data presented in (A)**. **Figure 4A**. Effect of suramin on the rate of *S. cervi *PFK-catalyzed reaction. Suramin concentrations were nil (○), 1 (Δ) and 5 nM (●). Mg^2+ ^concentration was constant (3.3 mM). The concentration of ATP was 0.1 mM. Enzyme concentration used was 10 μg/ml. Other conditions were same as described in Materials and Methods. **Figure 4B**. Hill plot of the data on the effect of suramin (0-10 nM) on the rate of *S. cervi *PFK-catalyzed reaction. The concentrations of F-6-P, ATP and Mg^2+ ^were 3.3, 0.1 and 3.3 mM. Other conditions were same as described in Materials and Methods. V_i _and V_0 _are rates of reaction in the presence and absence of suramin.

Several antifilarials have been tested for their effect on PFK of *S. cervi *PFK. The results are shown in Table 7. The strongest inhibition was observed with suramin, which was found to be effective in nM concentrations. To produce similar inhibitory effects, much higher concentrations of other compounds were required. The inhibition by suramin was of the non-competitive type and its K_i _value was found to be 1.1 ± 0.1 nM (Figure 4A). The Hill plot of the data at several suramin concentrations and a fixed F-6-P concentration (Figure 4B) showed a slope of 1.0, suggesting no cooperativity in the binding of suramin.

**Table 7:** Effect of antifilarial/anthelmintic compounds on the activity of *S.cervi *PFK

Compounds	% Residual Activity			
	
	Concentration (mM)			
	
	0	1	2	5
Levamisole	100	78	59	45

Diethylcitrate (DEC)	100	81	62	49

Cenperazine	100	73	51	40

Suramin	100	76	55	40

Compound 72/70*	100	57	39	29

The enzyme activity was assayed by preincubating it with the specific concentration of a desired compound for 15 min at 37°C and then residual PFK activity was monitored as described in Materials and Methods. Control denotes the enzyme activity measured in absence of any compound. *1-methyl-4-(piperidine-1-yl) carbonyl piperazine (Compound 72/70).

**Figure 5:**
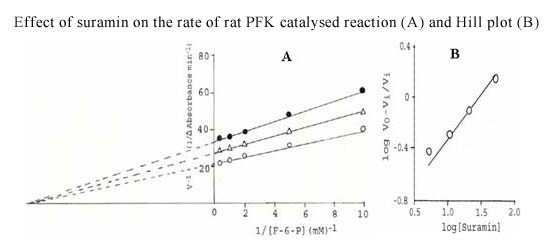
**Effect of suramin on the rate of rat liver PFK-catalyzed reaction (A) and Hill plot (B) of the data presented in (A)**. **Figure 5A**. Effect of suramin on the rate of rat liver PFK-catalyzed reaction. Suramin concentrations were nil (○), 1 (Δ) and 5 nM (●). Mg^2+ ^concentration was constant (3.3 mM). The concentration of ATP was 0.1 mM. Enzyme concentration used was 10 μg/ml. Other conditions were same as described in Materials and Methods. **Figure 5B**. Hill plot of the data on the effect of suramin (0-50 nM) on the rate of rat liver PFK-catalyzed reaction. The concentrations of F-6-P, ATP and Mg^2+ ^were 3.3, 0.1 and 3.3 mM. Other conditions were the same as described in Materials and Methods. V_i _and V_0 _are rates of reaction in the presence and absence of suramin.

Since, suramin exhibited a very strong inhibitory effect on the parasite enzyme; its effect was also studied on the activity of PFK isolated from a vertebrate tissue. For this purpose, PFK was partially purified from rat liver using standardized procedure [19] and the effect of suramin on this enzyme preparation was studied. Suramin was found to be inhibitory for the rat liver enzyme, but a comparatively higher concentration of this compound was required. The inhibition of rat liver PFK was of the non-competitive type (Figure 5A) and the K_i _value of suramin was found to be 40 ± 1 nM, which is about 40 times higher than that recorded for the parasite enzyme. The Hill plot of the data at several suramin concentrations and a fixed F-6-P concentration (Figure 5B) showed a slope equal to 1.0, suggesting no cooperativity in the binding of suramin to the mammalian enzyme.

## Discussion

### *S. cervi *PFK is cytosolic in nature

Studies on the sub-cellular localization of *S. cervi *PFK showed that it was mainly present in the soluble fraction of the homogenate of bovine filarial parasite. This is similar to the cytosolic localization of this enzyme observed in the vertebrates [5] and the parasite, *S. mansoni *[20]. However, in several Trypanosoma species, PFK has been shown to be present in a new type of sub-cellular membrane bound organelle termed as the glycosome, which contains many of the enzymes of glycolysis [21-23].

### Adults of *S. cervi *contain higher PFK activity than their microfilariae

Comparison of PFK activities in adult (male and female) and microfilarial stages of *S. cervi *showed highest activity of enzyme in adult (female/male) parasites. The specific activity of *S. cervi *PFK is close to that of the purified enzyme from human erythrocytes [24], white adipose tissues of rat [25] and *Onchocerca gutturosa *(adults) [26]. However, the specific activity of filarial enzyme was comparatively lower than the values reported for the enzyme purified from rabbit skeletal muscle [27], and erythrocytes [28], several other mammalian tissues [5], yeast [29] and some parasites such as *Echinococcus granulosus *[30] and *Brugia pahangi *adults [26]. The specific activity of *S. cervi *PFK was higher than that of *L. carinii *[31] and *S. mansoni *[26].

The enzyme from adult female worms has been purified over 100 fold with 30% recovery [2]. The purified PFK from *S. cervi *showed both similarities and differences when compared with the analogous enzyme from different sources [4].

### *S. cervi *PFK possess wide specificity towards utilization of nucleotides as phosphate group donors

A study of the nucleotide specificity for *S. cervi *PFK indicated that UTP, ATP and ATP were the best phosphate donors; ATP showing strongest inhibition at a higher concentration [4]. GTP, GDP and IDP were rather poor phosphate group donors and were also less inhibitory at higher concentrations. These results indicate that *S. cervi *PFK has a fairly wide specificity for various nucleotides as phosphate group donors. This is similar to the behaviour of the enzyme from vertebrate sources [32]. In the case of pig spleen enzyme, ATP, GTP and ITP are good phosphate group donors, whereas UTP and CTP are less effective [33]. Muscle enzyme can also use several derivatives of purine 5'-triphosphate [34].

### Modulation of *S. cervi *PFK by nucleotides depends on the concentration of its substrates

cAMP, AMP and ADP activated PFK from *S. cervi *at inhibitory concentrations of ATP (1.0 mM) and a low concentration of F-6-P (0.5 mM). These nucleotides also activate the enzyme from mammalian tissues [5,33-35] and a few trypanosomes [23,36]. In contrast, with the mammalian muscle PFK, it has been shown that the established inhibitors, such as citrate, activate the enzyme activity at low ATP or ITP concentrations while known activators, such as AMP, ADP, and cyclic AMP inhibit at low ATP or ITP concentrations [37].

### *S. cervi *PFK exhibits presence of a thiol group at its active site

*S. cervi *PFK was activated by some thiol compounds such as cysteine and β-ME, and inhibited by p-CMB. The partial reversal of p-CMB inhibition by addition of cysteine or β-ME suggests the functioning of -SH group at the active site of the enzyme molecule. The -SH groups have also been implicated in the catalytic activity of PFKs from some mammalian systems [5,38].

### *S. cervi *PFK is more sensitive towards antifilarials than the mammalian PFK

Among the different antifilarials tested, suramin was most effective in inhibiting PFK activity. Centperazine, DEC, levamisole and the compound 72/70 (synthesized at CDRI-Lucknow) inhibited this enzyme at higher (mM) concentrations. The inhibition of *S. cervi *PFK by suramin was non-competitive with respect to F-6-P. Suramin was also found to inhibit PFK of rat liver but at 40 times higher concentration (than that required for *S. cervi *enzyme), showing that the drug is comparatively more toxic to the parasite than the host. Suramin also has a strong inhibitory effect on lactic and malic dehydrogenases of *T. immitis *[39,40]; *Onchocerca volvulus *and *S. cervi *[41-44], protein kinase of *O. volvulus *[45] and *S. cervi *[46], β-D-glucosaminidase of *S. cervi *[47] and phosphatidylglycero-phosphate synthetase of *O. volvulus *and rat liver [48]. Furthermore, the results presented by Bronsvoort et al [49] indicated the potential of β-tubulin, the binding site of benzimidazoles, as a key molecular target for rational drug design of macrofilaricides. Very recently, Johnston et al [50] have reported that globomycin, a signal peptidase II (LspA) inhibitor in Gram-negative bacteria, is effective in reducing the motility and viability of adult *B. malayi in vitro*.

## Conclusions

Unlike other parasites, *S. cervi *PFK was present in its cytosolic fraction. The adult female *S. cervi *showed more enzyme activity than the microfilarial stage (Mf) of the parasite, suggesting presence of PFK in the musculature of the worm. The enzyme displayed a wide range of specificity towards utilization of nucleotides as phosphate group donors. However, the response of the enzyme to different nucleotides was dependent on the concentrations of F-6-P and ATP. The enzyme contains a thiol group at its active site and the inhibition of PFK by p-CMB could be protected to a significant extent by pretreatment with cysteine or β-ME. *S. cervi *PFK exhibited 40 times higher sensitivity towards suramin than that of mammalian PFK, thereby suggesting that this enzyme could be used as a potential chemotherapeutic target against filariasis.

## Materials and methods

### Materials

#### Parasite

Motile adult female worms (average length 6.0 ± 1.0 cm, average weight 35 ± 6.0 mg) and males (average length 4.0 ± 0.8 cm, average weight 6.0 ± 1.5 mg) of *S. cervi *were collected from the peritoneal folds of freshly slaughtered naturally infected Indian water buffaloes (Bubalus bubalis Linn.) at a local abattoir during early morning hours. The worms were brought to the laboratory in the Ringer's solution [51] within 2 h of slaughtering. The worms were thoroughly washed three-four times with lukewarm isotonic saline to remove the adhering contaminants. The worms were either frozen at -20°C until a week or used a fresh for this study.

#### Isolation of microfilariae (Mf)

The microfilariae (Mf) of *S. cervi *were collected by dissection of gravid females and by incubating the distal portion of the uteri (1 cm) for 3-4 h at 37 ± 1°C in Ringer's solution containing penicilline-G (1000 U/ml) and streptomycin sulfate (1000 U/ml). The Mf released into the medium were removed by low speed centrifugation and separated from the embryos and other tissues and washed twice with isotonic saline. The intact Mf could remain alive and active for 2 days at 4°C. The wet weight of one million Mf was about 36 mg.

#### Chemicals/Biochemicals

D-fructose-6-phosphate (F-6-P), adenosine-3', 5'-triphosphate (ATP), α-glycerophosphate dehydrogenase (GDH), D-fructose-1,6-diphosphate (FDP), triosephosphate isomerase (TPI), aldolase and phosphoenolpyruvate (PEP) were purchased from Sigma Chemical Co.-USA. Nicotinamide adenine dinucleotide reduced (NADH) was obtained from CSIR Centre of Biochemical Technology, New Delhi. Other reagents used were analytical grade.

#### Kreb's Ringer Bicarbonate (KRB) solution

This solution was prepared essentially according to the DeLuca and Cohen [52]. NaCl (9 g), KCl (0.42 g), glucose (0.50 g), NaCO_3 _(0.25 g) and CaCl_2 _(0.42 g) were added to the distilled water, made up to 1 L and the solution was sterilized by filtering through Millipore membrane filters (0.22 μm pore size).

### Methods

#### Preparation of tissue extract and purification of *S. cervi *PFK

PFK from adult female, male or Mf of *S. cervi *was isolated in the Tris-HCl buffer (50 mM, pH 8.0) containing ammonium sulfate (300 mM), β-mercaptoethanol (β-ME, 100 μM) and ATP (100 μM). A 10% (w/v) tissue homogenate of adult female parasite was prepared. The Mfs were treated with ultrasonic cell disrupter (Heat system, Ultrasonics Inc.Ltd., N.Y. W-220-F) on ice. The tissue extracts were centrifuged at 105,000 g for 60 min at 4°C and the cytosolic fractions were collected. The enzyme from adult female *S. cervi *was purified to electrophoretic homogeneity using very simple procedures and the activity was stabilized using suitable reagents [2].

#### Enzyme assay

*S. cervi *PFK was assayed using an enzyme coupled reaction method described by Racker [53] with slight modification as described by Sharma et al; [2]. In this method, we measured the formation of the D-fructose-1,6-diphosphate (FDP) using aldolase, TPI, GDH and NADH. The reaction mixture (3 ml) contained Tris HCl buffer (50 mM, pH 8.0), F-6-P (3.3 mM), ATP (0.1 mM), MgCl_2 _(3.3 mM), NADH (0.04 mM), GDH (0.66 Units/ml), TPI (5.6 Units/ml), aldolase (0.21 Units/ml) and suitable amount of enzyme protein (10-20 μg). The reaction was always started by adding substrate to the reaction mixture and the change in absorbance (oxidation of NADH to NAD^+^) after every 30 sec interval was measured spectrophotometrically at 340 nm. The extinction coefficient of NADH 6.22 × 10^3^M^-1^cm^-1 ^[54] was used to calculate the amount of oxidized pyridine nucleotide (NAD^+^). The three auxiliary enzymes such as aldolase, GDH and TPI were added in excess so that the overall reaction was governed by PFK activity present in the assay mixture. The concentration of Mg^2+ ^was kept higher than that of ATP (unless stated otherwise) for generating Mg-ATP complex (the substrate for the enzyme) and avoiding presence of free ATP molecules, which are known to be inhibitory in nature to PFK from other sources [5].

#### The reaction for PFK assay

**Figure 6:**
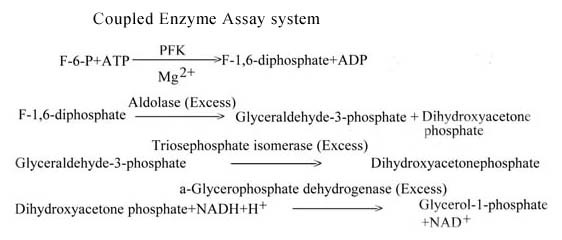
**Coupled enzyme assay system**. Schematic presentation of the biochemical reactions taking place during assay of the activity of *S. cervi *PFK. It is a coupled enzyme assay system in which the auxiliary enzymes have been used in excess so as to achieve overall rate of reaction being governed by PFK.

The scheme showing the reaction catalyzed by PFK in the assay system is displayed in Figure 6.

#### Determination of activation constant (K_a_)

The K_a _value for different activators were calculated from their corresponding double reciprocal plots using 1/V-V_0 _and 1/ [Nucleotide] on Y-and X-axes, respectively, where V and V_0 _represent the rates of PFK catalyzed reaction in the presence and absence of the effectors. The intersection point of the straight line at the negative abscissa of the X-axis was observed as -1/ K_a_.

#### Determination of the inhibition constant (K_i_)

The K_i _value for a non-competitive inhibitor was determined from the formula: Slope of inhibited reaction = K_m _/ V_max_.(1+ [Inhibitor]/K_i_). The Hill coefficient (n) value for the inhibitor has been determined from the slope of the Hill plot having Log V_0_-V_i _/ V_i _and Log [Inhibitor] values on Y-and X-axes. V_0 _and V_i _represent the rate of PFK catalyzed reaction in the absence and presence of the suramin.

## Competing interests

The author declares that they have no competing interests.

## Authors' contributions

The author (BS) approves the full manuscript.
